# Hypertext atlas of fetal and neonatal pathology

**DOI:** 10.1186/1746-1596-3-S1-S9

**Published:** 2008-07-15

**Authors:** Marta Ježová, Katarína Múčková, Ondřej Souček, Josef Feit, Pavel Vlašín

**Affiliations:** 1 Institute of Pathology, Masaryk University, Brno, Czech Republic; 2 Prenatal Diagnostic Center, Brno, Czech Republic

## Abstract

Hypertext atlas of fetal and neonatal pathology is a free resource for pregraduate students of medicine, pathologists and other health professionals dealing with prenatal medicine. The atlas can be found at . The access is restricted to registered users. Concise texts summarize the gross and microscopic pathology, etiology, and clinical signs of both common and rare fetal and neonatal conditions. The texts are illustrated with over 300 images that are accompanied by short comments. The atlas offers histological pictures of high quality. Virtual microscope interface is used to access the high-resolution histological images. Fetal ultrasound video clips are included. Case studies integrate clinical history, prenatal ultrasonographic examination, gross pathology and histological features. The atlas is available in English (and Czech) and equipped with an active index. The atlas is suitable both for medical students and pathologists as a teaching and reference tool. The atlas is going to be further expanded while keeping the high quality of the images.

## Introduction

Fetal pathology is a stand-alone discipline of pathology dealing with prenatal development, congenital anomalies and pregnancy failure. There has been dramatic advance in early prenatal diagnostics of fetal abnormalities that is now offered as a part of routine prenatal care. The affected couple mostly requests pregnancy termination. The main clinical utility of fetal autopsy is confirmation of prenatally diagnosed anomalies in the aborted fetus. The pathologist has a growing responsibility of reporting and documenting anomalies for the purposes of genetic counselling. There is also an increasing medical interest in identifying the causes of spontaneous fetal loss by means of fetal autopsy [1,2].

There has been no learning resource for the pregraduate students that would reflect the current knowledge and praxis of fetal and neonatal pathology. The standard textbooks offer only marginal depictions of placental pathology and common birth defects. Coping with this unsatisfactory situation we have prepared the multimedia atlas of fetal and neonatal pathology.

## Methods

Leica DMLA microscope with a set of PlanApo lenses (HC Fluotar 5/0.15, HC PlanApo 10/0.30, 20/0.50, 40/0.70, 100/1.30 and a Plan 2/0.07 lenses) equipped with the Nikon DMX-1200 digital camera is used to obtain image parts at the resolution at 1200 × 1020 pixels, 3 × 8 bit colour. Motorized stage (Merzhäuser) is automatically moved from one image to another. The system is controlled by Lucia DI (LIM, Prague). Images are further digitally processed and virtual slides are created.

Nikon Coolpix 8600 on a stand is used to take the macroscopic images.

## Results

The atlas can be found at . Registration is required to access the atlas. The atlas was uploaded in 2006 and has been continuously revised and expanded. The atlas exists in Czech and English version.

**Figure 1:**
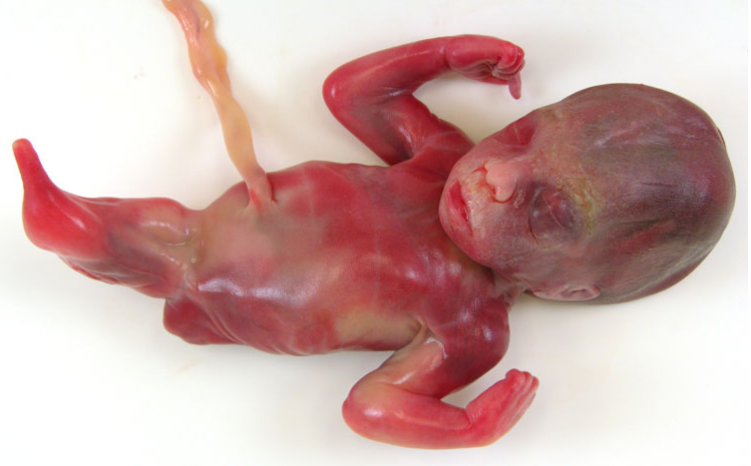
Sirenomelia, mermaid (malformation of the lower part of the body).

The users interface is based on the previous Hypertext Atlas of Dermatopathology (presented at  as well), sharing the internal structure and design. Concise texts summarize the gross and microscopic pathology and etiology, pathogenesis, clinical signs and prognosis. Plentiful macroscopic and histological images are included (see Figure 1). Virtual microscope interface is used to access the high-resolution histological images. The user can adopt the image detail, change magnification and change the basic size of the window up to full screen. Both the macroscopic and histological images are annotated and arrows pointing to the most important features can be activated.

The main chapters cover the principles and terminology of fetal maldevelopement, chromosomal abnormalities, teratogenic agents, malformation syndromes, congenital defects of the individual organ systems, pathology of twinning and pathology of placenta and umbilical cord.

The case studies seem to be quite useful for demonstrating complex malformation syndromes (e.g. sirenomelia) or pathogenetic pathways of some fetal conditions (e.g. twin to twin transfusion). The case studies integrate results of prenatal diagnostic tests, pathologic examination and genetic counselling. Ultrasound video clips have been included in the atlas recently. The user is encouraged to compare the gross pathology, histological image (if essential for final diagnosis) and prenatal video.

The atlas is equipped with an active index and a list of authors who contributed by texts or images.

## Discussion

Digital publication has many advantages, like (almost) unlimited capacity and easy maintenance and updating. Free on-line access is acceptable and comfortable for pre-graduate and post-graduate students who constitute the majority of the registered users.

The atlas currently provides a database of more than 300 macroscopic and histological images on fetal pathology. It is possible to be used as an image resource for teachers of pathology.

The atlas is under continuous development. New case studies, images and texts including fundamentals of neonatal pathology will be added in the future.

## Conclusion

The Hypertext Atlas of Fetal and Neonatal Pathology is a multimedia textbook for medical students and can be used by teachers of pathology as well. It is also a valuable reference tool for medical disciplines dealing with prenatal medicine. The access is at no cost but requires registration. The atlas is going to be further expanded while keeping the high quality of the images.
